# Generation, characterization, and validation of two human induced pluripotent stem cell lines from the peripheral blood of young and older adults

**DOI:** 10.1016/j.scr.2025.103670

**Published:** 2025-01-27

**Authors:** Dongli Yang, Jun Chen, Jerry H. Juratli, Andre Monteiro da Rocha, Allison Schley, Nadia R. Sutton

**Affiliations:** a Department of Medicine, Division of Cardiovascular Medicine, Vanderbilt University Medical Center Nashville TN USA; b Department of Internal Medicine, University of Michigan Ann Arbor MI USA; c Department of Biomedical Engineering, Vanderbilt University Nashville TN USA

**Keywords:** Aging, Stem cell, Epigenetic, Human induced pluripotent stem cell

## Abstract

Aging is a leading risk factor for the development of age-related diseases. However, how aging impacts human induced pluripotent stem cell (hiPSC) reprogramming, age-related epigenetic memory post-reprogramming, differentiation, and its potential applicability to cardiovascular regenerative medicine remains underexplored. We generated, characterized, and validated two hiPSC lines from human peripheral blood mononuclear cells (PBMCs) obtained from whole blood of young and older human donors. The two hiPSC lines expressed four pluripotency markers, have normal karyotypes and trilineage differentiation potential, and genetically match parental PBMCs. These lines are invaluable for regenerative medicine and exploring epigenetic-related molecular mechanisms that underlie aging and aging-related diseases.

## 1. Resource utility

How aging impacts human induced pluripotent stem cell (hiPSC) age-related epigenetic memory post-reprogramming, differentiation, and its potential applicability to regenerative medicine remains underexplored. The established two hiPSC lines, VUMCCVi001-A and VUMCCVi002-A, are an invaluable resource and offer a wide range of applications for regenerative medicine and basic research including age-related epigenetic memory post-reprogramming.

## 2. Resource details

Aging is a leading risk factor for the development of age-related diseases such as cardiovascular disease. (Sutton et al., 2023; Ali et al., 2024) However, how aging impacts hiPSC age-related epigenetic memory post-reprogramming, differentiation, and its potential applicability to cardiovascular regenerative medicine remains underexplored. There is an unmet need to develop invaluable resources for studying aging mechanisms to delay or reverse aging, thereby delaying or preventing development of cardiovascular diseases.

**Fig. 1.**
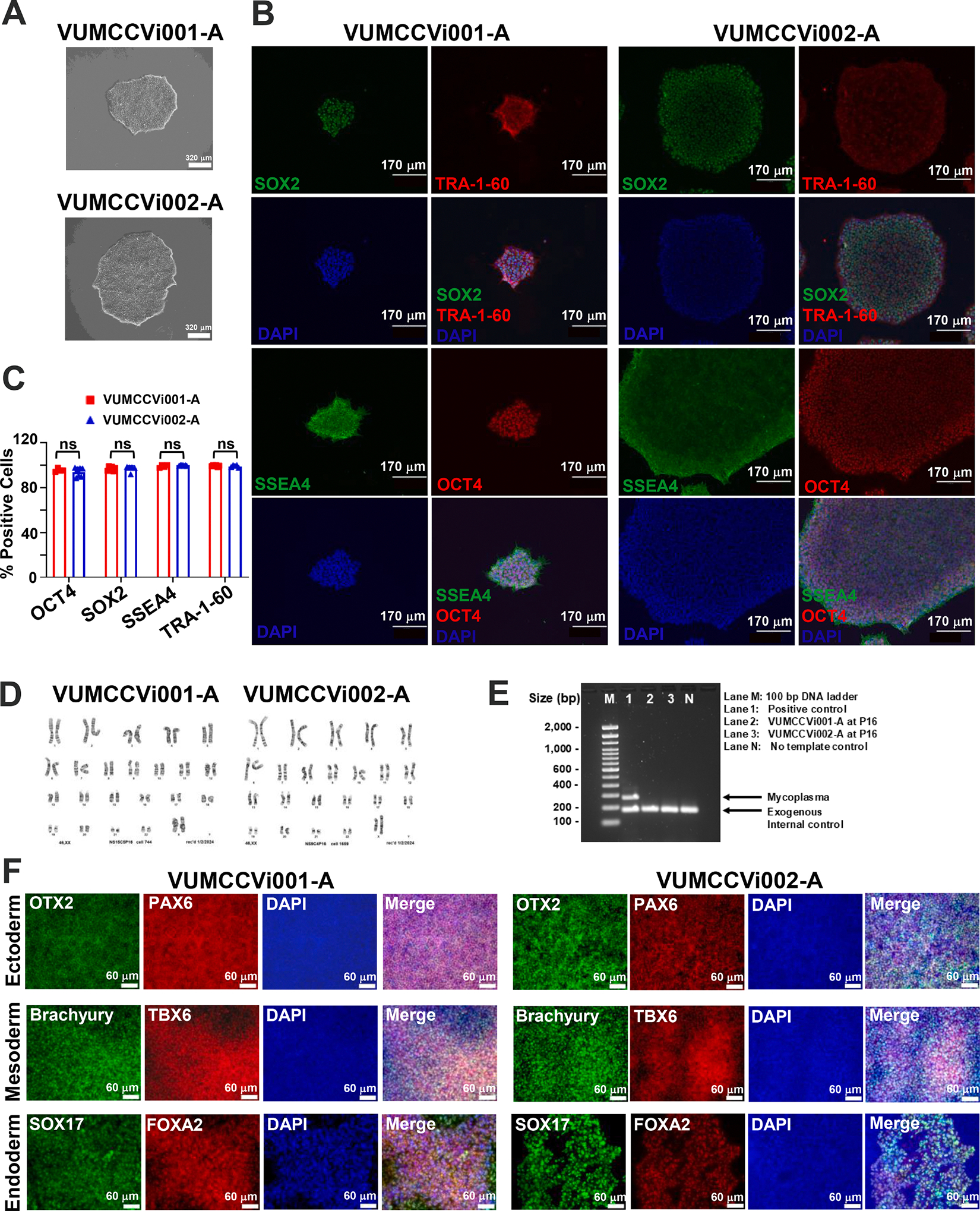
Characterization of two hiPSC lines VUMCCVi001-A and VUMCCVi002-A from young and older donors.

We generated two iPSC lines from two Caucasian females’ peripheral blood mononuclear cells (PBMCs) using Sendai virus (SeV)-based reprogramming vectors containing four Yamanaka factors (Oct4, Sox2, Klf4, and c-Myc) (Takahashi et al., 2007) and cultured them under feeder-free conditions. VUMCCVi001-A and VUMCCVi002-A were from a 25-year-old donor and an 84-year-old donor, respectively. SeV–based reprogramming was chosen because it is a non-integrating reprogramming method and generates iPSCs with lower incidence of karyotypic instability than episomal reprogramming. (Schlaeger et al., 2015) PBMCs were chosen as the donor cell source rather than dermal fibroblasts due to the ease and less invasive nature of obtaining PBMCs by venipuncture compared with dermal fibroblasts, which are obtained through a biopsy. The clonal iPSC lines VUMCCVi001-A and VUMCCVi002-A were established (Resource Table). Both iPSC lines displayed typical undifferentiated stem cell morphology (Fig. 1A) and expressed all four pluripotency markers: octamer-binding transcription factor 4 (OCT4), sex determining region Y-box 2 (SOX2), stage-specific embryonic antigen-4 (SSEA4), and tumor rejection antigen (TRA-1–60) at protein levels (Fig. 1B). Quantitative analysis showed that 93.9 % – 99.8 % cells express four pluripotency markers. Both lines showed comparable expression of all four pluripotency markers (*P* > 0.05) (Fig. 1C). Karyotype analysis demonstrated that both lines had normal diploid karyotypes and were female (46, XX) (Fig. 1D, Table 1). A 16-marker short tandem repeat (STR) analysis confirmed that VUMCCVi001-A and VUMCCVi002-A were identical to its own parental PBMCs, respectively (Table 1). Mycoplasma PCR testing showed that the 170 bp exogenous internal control was observed in all the samples, indicating the PCR testing was valid, and that the mycoplasma-specific 270 bp band was only detected in the positive control, not in both iPSC lines at passage 16 (P16) and the no template control (Fig. 1E, Table 1). Thus, both lines were mycoplasma-free. Both iPSC lines demonstrated trilineage differentiation potential with expression of three germ layer markers: OTX2, PAX6 (ectoderm markers); Brachyury, TBX6 (mesoderm markers) and SOX17, FOXA2 (endoderm markers) as demonstrated by fluorescent immunocytochemistry (ICC) (Fig. 1F, Table 1). As the reprogramming vector used, SeV, is an RNA virus, RT-PCR analysis was used to confirm the loss of reprogramming vectors (Resource Table). SeV genome-specific primers as well as internal control glyceraldehyde 3-phosphate dehydrogenase (GAPDH)-specific primers are listed in Table 2. Reprogramming vectors were not detected in both lines at P16, although they were detected at early passages (P1, P3 or P7) (Supplementary Fig. 1A). A 131 bp internal control GAPDH band was detected at all passages, but not in the no template control (Supplementary Fig. 1B). Thus, both iPSC lines are vector-free.

## 3. Materials and methods

### 3.1. PBMC isolation

The peripheral whole blood was collected and centrifuged at room temperature, and the layer containing PBMCs was collected, washed, and counted. The PBMCs were cryopreserved until use.

### 3.2. Reprogramming and iPSC culture

Frozen PBMCs were thawed, cultured, and then reprogrammed into hiPSCs using CytoTune^™^-iPS 2.0 Sendai Reprogramming Kit (Invitrogen^™^, Cat# A16517) that contains four Yamanaka factors (Oct3/4, Sox2, Klf4, and c-Myc) under feeder-free conditions following the manufacturer’s instructions. A complete mTeSR1 (STEMCELL Technologies Inc, Cat# 85850) was used to maintain iPSCs. Ten μM of ROCK inhibitor (Y27632, STEMCELL Technologies Inc, Cat# 72302) was included in the medium when passaging iPSCs. The medium was changed every day. At ~ 70–85 % confluency, iPSC cells were lifted using Versene Solution (Gibco^™^, Cat# 15040066), seeded on hESC-Qualified Matrigel (Corning, Cat# 354277)-coated plates at split ratios of 1:6 −1:12 and cultured in a humidified incubator at 37 °C with 5 % CO_2_.

### 3.3. Trilineage differentiation

*In vitro* trilineage differentiation was conducted for cells using STEMdiff^™^ Trilineage Differentiation Kit (STEMCELL Technologies, Cat# 05230) according to the manufacturer’s instructions.

### 3.4. Fluorescent immunocytochemistry

Fluorescent immunocytochemistry (ICC) of four pluripotency markers and six differentiation markers was performed for both lines. The Pluripotent Stem Cell 4-Marker ICC Kit (Invitrogen^™^, Cat# A24881) was used for staining four pluripotency markers (Table 2) following the manufacturer’s instructions. For six differentiation markers, fluorescent ICC staining procedures (Khokhar et al., 2024) were performed for cells at P16 with primary and secondary antibodies listed in Table 2. Cells were imaged with a fluorescence microscope (ECHO REVOLVE R4 Upright & Inverted Microscope).

### 3.5. RNA isolation and RT-PCR analysis

RT-PCR Analysis was used to confirm the loss of reprogramming vectors. Briefly, total RNAs were extracted from each iPSC line at passages up to 16 using RNeasy-mini kit (Qiagen), and cDNAs were synthesized using the SuperScript^™^ VILO^™^ cDNA Synthesis Kit (Invitrogen^™^, Cat# 11754050). PCR was performed by mixing cDNA, SeV-specific primers (Table 2) and AccuPrime^™^ Pfx SuperMix (Invitrogen^™^, Cat# 12344040) with initial denaturation at 95 °C for 5 min and cycled 35 times (15 s at 95 °C, 30 s at 55 °C, 30 s at 68 °C), followed by a 5-minute extension at 68 °C. The housekeeping gene GAPDH served as an internal control. The RT-PCR products were analyzed by 2.0 % agarose gel electrophoresis.

### 3.6. Karyotype analysis

Karyotype analysis was performed at P16 and evaluated by Genetics Associates Inc. (Nashville, TN, USA) using the Q-banding technique.

### 3.7. Genomic DNA isolation and short tandem repeat (STR) analysis

Genomic DNA was extracted from two iPSC lines at P16 and their parental PBMCs using the DNeasy Blood & Tissue Kit (Qiagen, Cat #69504). STR analysis was performed by IDEXX BioAnalytics (Columbia, MO, USA).

### 3.8. Mycoplasma PCR Detection

The mycoplasma testing was performed with the Myco-Sniff-Valid^™^ Mycoplasma PCR Detection Kit (MP Biomedicals^™^, Cat# 093050301) following manufacturer’s instructions. In addition to detecting the mycoplasma-specific 270 bp band, an exogenous internal control (170 bp) was provided with this kit to help differentiate false negatives due to PCR inhibition or erroneous PCR tests.

## Supplementary Material

Supplementary data

Appendix A. Supplementary data

Supplementary data to this article can be found online at https://doi.org/10.1016/j.scr.2025.103670.

## Figures and Tables

**Table 1:** Characterization and validation procedures performed on two human induced pluripotent stem cell lines.

Classification	Test	Result	Data

**Morphology**	Photography bright field	Visual record of the line: normal	Fig. 1 *panel A*
**Phenotype**	Qualitative analysis: Immunocytochemistry (ICC)	Positive expression of 4 pluripotency markers: OCT4, SOX2, SSEA4 and TRA-1–60	Fig. 1 *panel B*
	Quantitative analysis: ICC counting	**VUMCCVi001-A** OCT4^+^ cells: 95.5 % ± 1.6 % (n = 4) SOX2^+^ cells: 96.9 % ± 1.9 % (n = 6) SSEA4^+^ cells: 99.0 % ± 1.2 % (n = 4) TRA-1–60^+^ cells: 99.3 % ± 0.7 % (n = 6) **VUMCCVi002-A** OCT4^+^ cells: 93.9 % ± 4.1 % (n = 7) SOX2^+^ cells: 96.8 % ± 2.7 % (n = 5) SSEA4^+^ cells: 99.8 % ± 0.3 % (n = 7) TRA-1–60^+^ cells: 99.1 % ± 1.0 % (n = 5)	Fig. 1 *panel C*
**Genotype**	Karyotype (G-banding) and resolution	Normal karyotype: 46, XX for both lines Resolution: 450–500	Fig. 1 *panel D*
***mtDNA analysis* (IF APPLICABLE)**	N/A	N/A	N/A
**Identity**	Microsatellite PCR (mPCR) OR STR analysis	N/A	N/A
		16 loci tested: 100 % Matching	Submitted in archive with journal
**Mutation analysis (IF APPLICABLE)**	Sequencing	N/A	N/A
	Southern Blot OR WGS	N/A	N/A
**Microbiology and virology**	Mycoplasma	Mycoplasma testing by PCR: Negative	Fig. 1 *panel E*
**Differentiation potential**	Directed differentiation	Positive expression of three germ layer markers demonstrated at protein levels List of markers: Ectoderm: OTX2, PAX6 Mesoderm: TBXT (Brachyury), TBX6 Endoderm: SOX17, FOXA2	Fig. 1 *panel F*
**Donor screening (OPTIONAL)**	HIV 1 + 2 Hepatitis B, Hepatitis C	N/A	N/A
**Genotype additional info (OPTIONAL)**	Blood group genotyping	N/A	N/A
	HLA tissue typing	N/A	N/A

**Table 2:** Details of Reagents Utilized for Stem Cell Line Validation.

	Antibodies used for immunocytochemistry			
	
	Antibody	Dilution	Company Cat #	RRID

Pluripotency Marker	Rat Anti-Human SOX2 (Part of PSC 4-Marker ICC Kit)	1:100	Invitrogen^™^ Cat# A24881	RRID: AB_2889933
Pluripotency Marker	Mouse Anti-Human TRA-1–60 (Part of PSC 4-Marker ICC Kit)	1:100	Invitrogen^™^ Cat# A24881	RRID: AB_2889933
Pluripotency Marker	Mouse Anti-Human TRA-1–60 (Part of PSC 4-Marker ICC Kit)	1:100	Invitrogen^™^ Cat# A24881	RRID: AB_2889933
Pluripotency Marker	Mouse Anti-Human TRA-1–60 (Part of PSC 4-Marker ICC Kit)	1:200	Invitrogen^™^ Cat# A24881	RRID: AB_2889933
Differentiation Marker: Ectoderm Marker	Goat Anti-OTX2	1:50 (4 μg/mL)	R&D Systems Cat# AF1979	RRID: AB_2157172
Differentiation Marker: Ectoderm Marker	Rabbit Anti-PAX6	1:100 (2.5 μg/mL)	Invitrogen^™^ Cat# 426600	RRID: AB_2533534
Differentiation Marker: Mesoderm Marker	Goat Anti-Brachyury	1:50 (4 μg/mL)	R&D Systems Cat# AF2085	RRID: AB_2200235
Differentiation Marker: Mesoderm Marker	Rabbit Anti-TBX6	1:50 (10 μg/mL)	Invitrogen^™^ Cat# PA535102	RRID: AB_2552412
Differentiation Marker: Endoderm Marker	Goat Anti-SOX17	1:50 (4 μg/mL)	R&D Systems Cat# AF1924	RRID:AB_355060
Differentiation Marker: Endoderm Marker	Rabbit Anti-FOXA2	1:200 (2.5 μg/mL)	Invitrogen^™^ Cat# 701698	RRID: AB_2576439
Secondary antibody	Alexa Fluor^™^ 488 Donkey Anti-Rat (Part of PSC 4-Marker ICC Kit)	1:250	Invitrogen^™^ Cat# A24881	RRID: AB_2889933
Secondary antibody	Alexa Fluor^™^ 594 Goat Anti-Mouse IgM (Part of PSC 4-Marker ICC Kit)	1:250	Invitrogen^™^ Cat# A24881	RRID: AB_2889933
Secondary antibody	Alexa Fluor^™^ 488 Goat Anti-Mouse IgG3 (Part of PSC 4-Marker ICC Kit)	1:250	Invitrogen^™^ Cat# A24881	RRID: AB_2889933
Secondary antibody	Alexa Fluor^™^ 594 Donkey Anti-Rabbit (Part of PSC 4-Marker ICC Kit)	1:250	Invitrogen^™^ Cat# A24881	RRID: AB_2889933
Secondary antibody	Alexa Fluor 488 Donkey Anti-Goat IgG	1:250	Invitrogen^™^ Cat# A-11055	RRID: AB_2534102
Secondary antibody	Alexa Flour 594 Donkey Anti-Rabbit IgG	1:250	Invitrogen^™^ Cat# A-21207	RRID:AB_141637
	Primers Target	Size of band	Forward/Reverse primer (5′-3′)	
Sendai Virus (SeV) Vectors (RT-PCR)	SeV genome	181 bp	GGATCACTAGGTGATATCGAGC/ACCAGACAAGAGTTTAAGAGATATGTATC	
Housekeeping Gene (RT-PCR)	GAPDH	131 bp	GTCTCCTCTGACTTCAACAGCG/ACCACCCTGTTGCTGTAGCCAA	

**Resource Table:** 

Unique stem cell lines identifier	1) VUMCCVi001-A 2) VUMCCVi002-A
Alternative name(s) of stem cell lines	1) NS15C5 2) NS9C4
Institution	Department of Internal Medicine, Division of Cardiovascular Medicine, Vanderbilt University Medical Center, Nashville, TN, USA
Contact information of distributor	Dr. Nadia R. Sutton: nadia.sutton@vumc.org Dr. Dongli Yang: dongli.yang@vumc.org
Type of cell lines	iPSC
Origin	human
Additional origin info required *for human ESC or iPSC*	Age: 25 (VUMCCVi001-A), 84 (VUMCCVi002-A)Sex: Female (both lines)Ethnicity: Caucasian (both lines)
Cell Source	PBMCs (both lines)
Clonality	Clonal
Method of reprogramming	Sendai virus reprogramming
Genetic Modification	No
Type of Genetic Modification	N/A
Evidence of the reprogramming transgene loss (including genomic copy if applicable)	RT-PCR
Associated disease	1) VUMCCVi001-A: none2) VUMCCVi002-A: severe aortic stenosis, hypertension, dyslipidemia
Gene/locus	N/A
Date archived/stock date	1) VUMCCVi001-A: 2022 2) VUMCCVi002-A: 2022
Cell line repository/bank	https://hpscreg.eu/cell-line/VUMCCVi001-A https://hpscreg.eu/cell-line/VUMCCVi002-A
Ethical approval	The research protocol including generation of the iPSC lines was approved by the University of Michigan (IRB HUM00175056) and Vanderbilt University Medical Center (IRB #230567, IBC# VBMR-0535).
