# Supplementary material for: Generation, characterization, and validation of two human induced pluripotent stem cell lines from the peripheral blood of young and older adults
Source: Stem Cell Res. Author manuscript; Available in PMC 2025 Aug 1. (PMC12315593; doi:10.1016/j.scr.2025.103670)
Supplement: Supplementary data [file NIHMS2097886-supplement-Supplementary_data.pdf]

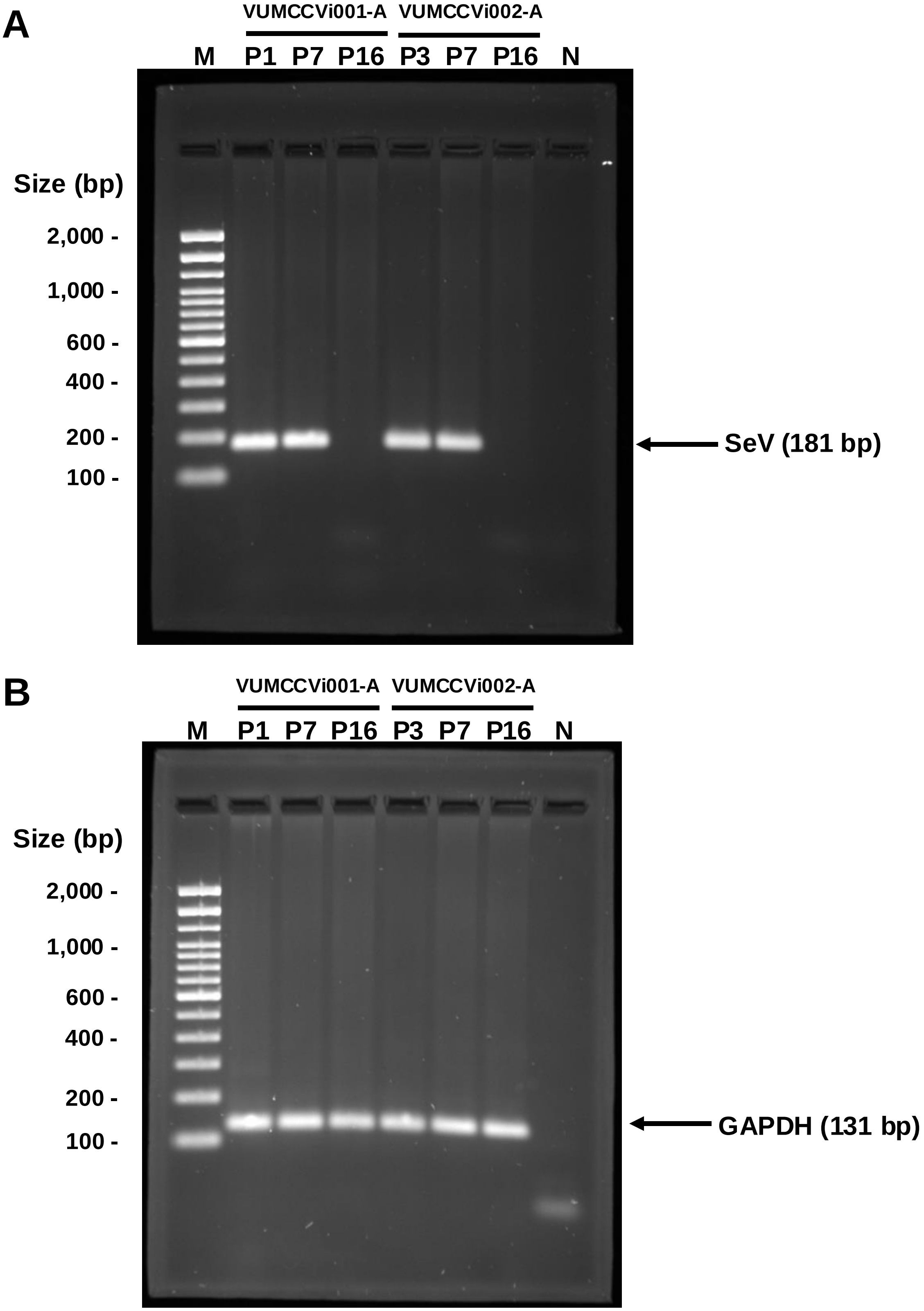

Supplementary Figure 1. Loss of reprogramming vectors in two hiPSC lines VUMCCVi001-A and VUMCCVi002-A from young and older donors. RNAs extracted from the indicated hiPSC lines at different passages (P1, P3, P7 or P16) were subjected to RT-PCR using reprogramming vector Sendai virus (SeV)-specific primers. The housekeeping gene, glyceraldehyde 3-phosphate dehydrogenase (GAPDH), served as an internal control. Primers are listed in Table 2. RT-PCR products were resolved on 2.0% agarose gel. Lane M: 100 bp DNA Ladder (Invitrogen™, Cat# 15628019); Lane N: No template control.
